# OR5-001 – Characterization of tonsil infiltration in PFAPA

**DOI:** 10.1186/1546-0096-11-S1-A94

**Published:** 2013-11-08

**Authors:** S Chiesa, R Caorsi, F Penco, A Bertoni, S Borghini, A Sementa, A Omenetti, F Bellora, R D'Agostino, A Martini, M Gattorno

**Affiliations:** 1 Laboratory of Immunology and Rheumatic Diseases, U.O. Pediatria II, G. Gaslini Institute, Genoa, Italy; 2 Laboratory of Molecular Genetics, G. Gaslini Institute, Genoa, Italy; 3 Anatomic Pathology, G. Gaslini Institute, Genoa, Italy; 4 Medicina Sperimentale, University of Genoa, Genoa, Italy; 5 U.O. Otorinolaringoiatria, G. Gaslini Institute, Genoa, Italy

## Introduction

The syndrome of periodic fever, aphthous stomatitis, pharyngitis, and cervical adenitis (PFAPA) is the most common periodic fever disease in young children. The etiology of this disorder is still unknown. Palatine tonsils are sites where innate immunity leads to onset of the adaptive immunity, mediated by B and T lymphocytes. Three families of pathogen sensors mediate the recognition of microbes: Toll-like receptors (TLRs), NOD-like receptors (NLRs) and RIG-I-like receptors (RLRs). The interplay of these receptors ensures the efficient coordination of innate immune responses.

## Objectives

We aimed to investigate differences in leukocyte subpopulations and innate receptors gene expression of palatine tonsil cells from patients with PFAPA in order to understand the pathogenesis of this inflammatory condition.

## Methods

We have collected tonsil tissue from 2 groups of pediatric patients undergoing tonsillectomy: PFAPA patients (n=20) and patients who had indication of recurrent bacterial tonsillitis (control group, CG) (n=16). We have performed staining of subpopulations on tonsil cells and tissues using, respectively, flow cytometry and immunohistochemistry assays . We have analyzed TLRs, NLRs, and RLRs gene expression profiles by quantitative real-time RT-PCR.

## Results

Immunohistochemistry analysis has shown preservation of tonsillar architecture without any specific chronic inflammation with respect to GC. FACS analysis has demonstrated a higher number of naïve and a significantly lower percentage of effector memory CD4^+^ and CD8^+^ T cells in PFAPA patients compared to CG. Remarkably, we have observed a considerably recruitment of NK cells in tonsils of PFAPA patients with respect to CG. In particular, we have detected a significant expansion of CD56^+^CD16^-^ and CD56^+^CD16^+^ NK cell subsets when compared to CG. A detailed characterization of NK activating receptors and NK cell functions in PFAPA tonsils is still in progress. Finally, in PFAPA patients we have revealed a significant increase in the gene expression of NALP1 and NALP3 when compared to CG.

## Conclusion

These results indicate a possible involvement of NK cells and of innate receptors in pathogenesis of PFAPA supporting the crucial role of the innate immunity. Nonetheless, the high numbers of undifferentiated naïve T cells in PFAPA patients suggest that adaptive immune responses might be implicated in these autoinflammatory disorders. Tonsillectomy seems to be an effective treatment for PFAPA syndrome.

## Disclosure of interest

None declared

